# *Didymocarpus puhoatensis* (Gesneriaceae), a new species from Vietnam

**DOI:** 10.3897/phytokeys.94.21650

**Published:** 2018-01-29

**Authors:** Xin Hong, Zhen-Long Li, Stephen Maciejewski, Fang Wen, Truong Van Do

**Affiliations:** 1 School of Resources and Environmental Engineering, Anhui University, CN–230601, Hefei, China; 2 The Gesneriad Society, Inc. 1122 East Pike Street, PMB 637, Seattle, Washington, USA; 3 Gesneriad Conservation Centre of China, Guangxi Key Laboratory of Plant Conservation and Restoration Ecology in Karst Terrain, Guangxi Institute of Botany, Guangxi Zhuang Autonomous Region and Chinese Academy of Sciences, CN-541006 Guilin, China; 4 Vietnam National Museum of Nature, Vietnam Academy of Science & Technology, 18 Hoang Quoc Viet, Hanoi, Vietnam

**Keywords:** Didymocarpus, Gesneriaceae, plant taxonomy, Vietnam

## Abstract

*Didymocarpus puhoatensis*, a new species from Vietnam is described and illustrated with photographs. The new species is morphologically similar to *D. brevicalyx* and *D. epithemoides*, but can be easily distinguished by a combination of characters. So far, five species have been recorded in the genus *Didymocarpus* from Vietnam.

## Introduction

The delimitation of the genus *Didymocarpus* Wallich (1819: 378) has varied considerably over time (Burtt 1998, Weber et al. 2000, 2011, Möller et al. 2011, Möller and Clark 2013, Li et al. 2015). Now approximately 70 species range from northwest India, eastwards through Nepal, Bhutan, northeast India, Burma (Myanmar), to southern China, Vietnam, Laos, Cambodia, Thailand, the Malay Peninsula and northwards to Sumatra (Weber and Burtt 1998, Weber et al. 2000, Möller et al. 2016). Only three species of this genus were found in Vietnam before 2012, then *D. kerrii* and *D. purpureobracteatus* were respectively reported as new record species for the flora of Vietnam (Phuong et al. 2012, 2014). *Didymocarpus bonii* [= *Calcareoboea bonii*], is now a synonym of *Petrocodon bonii* (Weber et al. 2011). So now there are four species of *Didymocarpus* recorded in Vietnam: *D. kerrii*, *D. pulcher*, *D. poilanei* and *D. purpureobracteatus*.

During a floristic expedition to northern Vietnam in 2015, the authors observed a population of an interesting Gesneriaceae in Pu Hoat Nature Reserve, Nghe An province, Vietnam. It was confirmed that it is member of the genus *Didymocarpus* based on its disc-like stigma (Wang et al. 1998). Over the past two years, the living plants were monitored in the field and an ecological survey was carried out by the co-author in Vietnam and in the nursery of Gesneriads Conservation Centre of China (GCCC) in China.

After thorough comparisons of diagnostic morphological and anatomical features of similar taxa from China, Vietnam, Thailand and adjacent regions (Kiew 1990, Hilliard and Burtt 1995, Wang et al. 1998, Burtt 1998, 1999, Weber et al. 2000, Hilliard 2001, Nangngam and Maxwell 2013, Nangngam and Middleton 2014, Phuong et al. 2014), it is concluded that, as its morphological characters do not fit any known species, it is a new species to science and accordingly described herein. Its morphological characters are compared with the closely related species: *D. brevicalyx* Nangngam & D.J. Middleton (2014: 35) and *D. epithemoides* B.L. Burtt (2001: 92). Therefore, there are five species of the *Didymocarpus* recorded in Vietnam.

## Material and methods

Measurements and morphological character assessments of the putative new species were performed and described using specimens’ work by the current authors, living material observed in the field and also those cultivated at the Gesneriad Conservation Centre of China. All available specimens of Southeast Asian *Didymocarpus* kept in the following herbaria were examined: E, GH, HN, IBK, K, KUN, MO, PE, PH, US and VNMN. The images of type specimens were also obtained from Tropicos (http://www.tropicos.org), JSTOR Global Plants (http://plants.jstor.org) and the International Plant Names Index (http://www.ipni.org). All morphological characters were studied under dissecting microscopes and are described using the terminology presented by Wang et al. (1990, 1998).

## Taxonomic treatment

### Didymocarpus puhoatensis

Taxon classificationPlantaeLamialesGesneriaceae

X.Hong & F.Wen sp. nov.

urn:lsid:ipni.org:names:77175491-1

Figures 1 , 2

#### Diagnosis.

Although it is morphologically similar to *D. brevicalyx*, it differs by stem densely pubescent, orbicular purple bracts, apices of calyx lobes obtuse, filaments glabrous, staminodes 2; and also similar to *D. epithemoides*, but differs from the latter in having purple calyx, funnel-form corolla, 4–5 cm long, glabrous, dark purple-blackish, ovary glandular puberulent.

#### Type.

VIETNAM. Nghệ An Province: Quế Phong, Thông Thụ, Pu Hoat Nature Reserve (Khu Bảo tồn thiên nhiên Pù Hoạt), 19°52'30.5"N, 104°56'15.1"E, alt. 390 m, 18 July 2014, flowering, *Truong Van Do et al. VNM-CN439* (holotype: IBK; isotype: VNM).

#### Description.

Deciduous, perennial, lithophytic herb, 10–30 cm tall. *Stems* erect, single, sparsely puberulent to glabrescent, the upper, leaf-bearing part and young stems densely covered with whitish multicellular eglandular hairs. *Dry season* plants unknown. *Rainy season* leaves opposite, anisophyllous; petioles terete, 0.5–2.5 cm long, densely covered with multicellular eglandular hairs as on the stems; blades asymmetrically ovate, 6–10 cm long, 5–8 cm wide, apex bluntly acute, base slightly oblique, obtuse-cuneate, margin finely serrate or finely doubly serrate, papery, upper surface densely covered with whitish multicellular eglandular hairs, green, lower surface sparsely covered with hairs as on upper surface, pale green, venation pinnate, secondary veins 4–8 on each side of midrib, mostly opposite sometime alternate, obscure above, prominent beneath, covered with whitish multicellular eglandular hairs. *Inflorescences* terminal or from the upper leaf axils, cymose, ca. 12 cm long, 4–10 (–30) flowered; peduncles slender, 6–10 cm long, light green, sparsely covered with multicellular glandular hairs; pedicels 1–1.5 cm long, pale green, with indumentum as on the peduncle. *Bracts* paired, orbicular, ca. 5 mm long and wide, green to pale purple, glabrous. *Calyx* consisting of a tube and shallowly 5-lobed margin, symmetrical, campanulate, 6 mm long, glabrous, somewhat tawny to pinkish purple, tube ca. 5 mm long, 3 mm in diameter; lobes triangular, ca. 1 mm long, ca. 2 mm wide, apices obtuse. *Corolla* funnelform, 4–5 cm long, glabrous outside, blackish purple, becoming light purple at base; tube ca. 3.5 cm long, base narrow, ca. 2 mm wide, dilated and slightly ventricose towards the throat; widest at throat, diameter ca. 9 mm; corolla bi-lipped, lobes suborbicular; lower lip 3-lobed, ca. 8 mm long, 6 mm wide, more or less equal; upper lip 2-lobed, ca. 4 mm long and wide. Fertile stamens adnate to corolla ca. 1.5 cm from base; anther locules, ca. 2.5 mm long, ca. 1.5 mm wide, densely covered with brownish multicellular eglandular hairs; filaments slender, ca. 1 cm long, white, glabrous; staminodes 3, reduced to thin filaments, lateral ones 3 mm long, the other one 1 mm long, glabrous, adnate to corolla ca. 1 cm from base. *Disc* cylindrical, ca. 2 mm long, margin irregular. *Pistil* ca. 2–3 cm long; ovary narrowly linear, ca. 2 cm long, sparsely glandular puberulent, base reddish, with purple tinge towards stigma and apex green; stigma 1, peltate, concave, papillose, cream. *Capsules* unknown.

**Figure 1.**
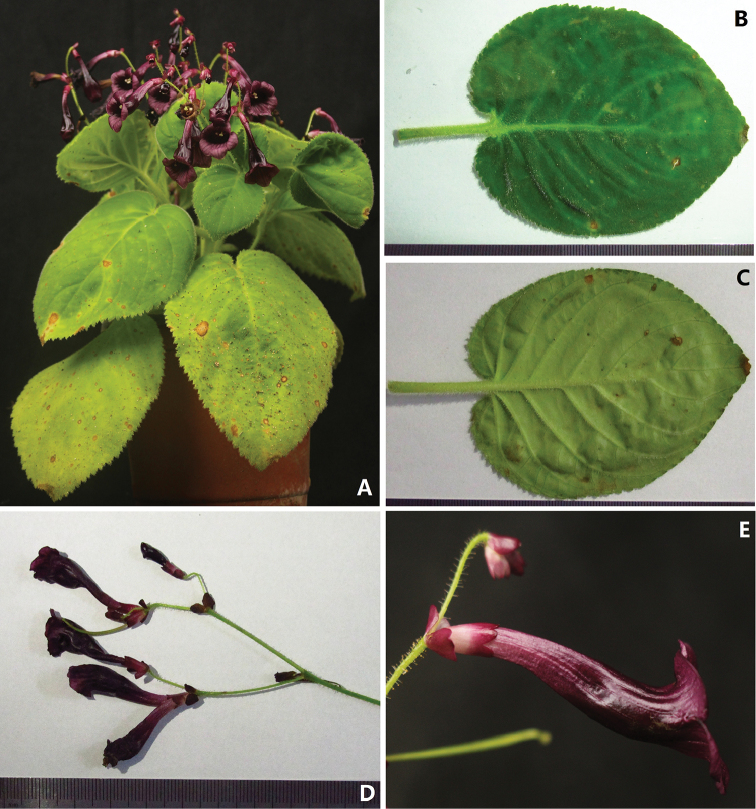
*Didymocarpus puhoatensis* X.Hong & F.Wen **A** Habitat **B** Adaxial surface view of leaf blade **C** Adaxial surface view of leaf blade **D** Cyme with flowers, showing the bracts **E** Lateral view of corolla, showing the calyx consisting of a tube.

**Figure 2.**
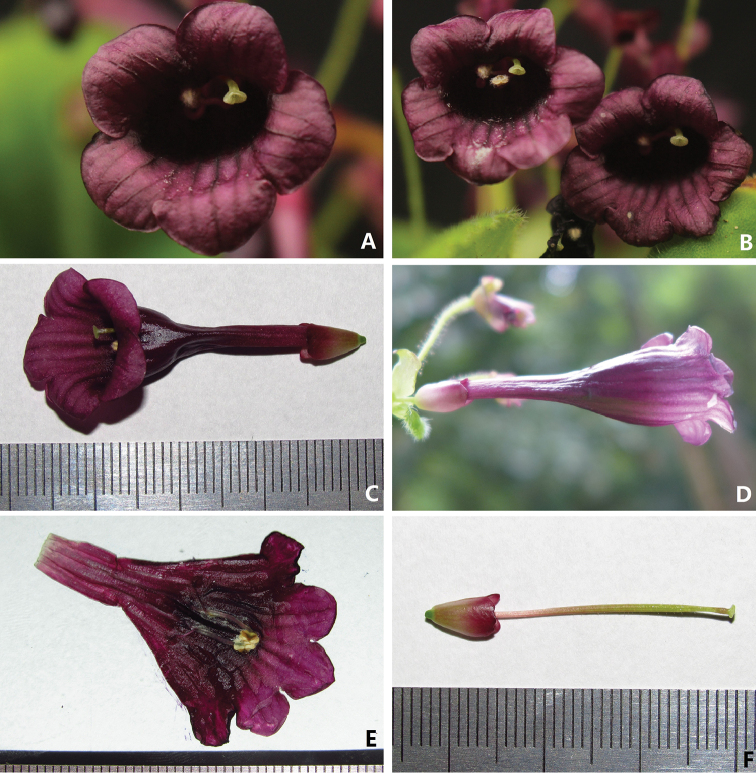
Flower of *Didymocarpus puhoatensis* X.Hong & F.Wen **A–B** Frontal view of corolla, showing the disc-like stigma **C** Top view of corolla **D** Upward view of corolla **E** Opened corolla, showing stamens and staminodes **F** Pistils without corolla.

#### Phenology.

It flowers and fruits from June to September.

#### Etymology.

The specific epithet is derived from the type locality, Pu Hoat Nature Reserve, Nghệ An Province, Vietnam.

#### Distribution and habitat.

This new taxon is an endemic species from Pu Hoat Nature Reserve of Vietnam. The species grows on limestone rocks in tropical monsoon forest with sufficient seasonal run-off water, at an elevation of 390 m a.s.l. It distributes much lower in altitude and the habitat is much hotter and more humid than other species with stems of the genus.

#### Preliminary conservation assessment.

As population information of *Didymocarpus puhoatensis* is still unclear, it is not appropriate to make an assessment of the extinction risk faced by this new taxon. Thus, the category of Data Deficient (DD) should be appropriate, according to IUCN (2016) criteria. Fortunately, the known habitat of the species is protected as part of a nature reserve. Besides prolonged droughts and illegal logging in the area, there are other potential risks to the persistence of this new species.

##### Key to the species of *Didymocarpus* in the Vietnam

**Table:** 

1	Ovary smooth	**2**
–	Ovary hair	**4**
2	Corolla outer hair; leaf blade ovate or elliptic, ca. 4–10 × 2–6 cm	***D. pulcher***
–	Corolla outer smooth; leaf blade near round or broadly elliptic, ca. 1–4 × 1–4 cm	***D. kerrii***
4	Base of leaf often axisymmetric; bract lanceolata, ca. 2–3 mm long; sepal tube ca. 3 mm long, sepal lobed ca. 1–1.5 mm long	***D. poilanei***
–	Base of leaf often oblique; bract elliptic ovate or orbicular, ca. 3–8 mm long; sepal tube ca. 8–9 mm long, sepal lobed ca. 2–3 mm long	**5**
5	Peduncle 4–10 cm, glabrous; bracts connate at base; calyx 1–1.2 cm; corolla 3–4 cm, purple to pinkish purple	***D. purpureobracteatus***
–	peduncles 6–10 cm, glandular hairs; bracts separate; calyx 6 mm; corolla 4–5 cm, blackish purple	***D. puhoatensis***

## Discussion

It is morphologically similar to *D. brevicalyx* and *D. epithemoides* in having the calyx consisting of a tube, similar shape and colour of corolla, both morphological affinities being distributed in Thailand. However, *D. puhoatensis* can be clearly differentiated from both by several characters. The major differences between the species are outlined in Table 1.

**Table 1.** Diagnostic characters for *Didymocarpus puhoatensis* sp. nov. and its relatives.

Character	*Didymocarpus puhoatensis*	*D. brevicalyx*	*D. epithemoides*
**Indumentum of Stem**	densely pubescent	densely glandular pubescent	densely pubescent
**Bracts**	orbicularc. 5 mm long and wide	triangular c. 2 mm long and 1.5 mm wide	orbicular, 5 mm long and wide
**Calyx**	lobes apices obtuse, purple	lobes apices acute, reddish	lobes apices rounded, violet
**Corolla**	funnelform, 4–5 cm long, glabrous, dark purple-blackish	funnelform, 4.5 cm long, glabrous, dark purple-blackish	salverform, 3–3.5 cm long, glandular pubescent outside, dark violet
**filaments**	glabrous	gland-tipped hairs on the upper part	glabrous
**Indumentum of ovary**	sparsely glandular puberulent	densely glandular pubescent	glabrous

## Supplementary Material

XML Treatment for Didymocarpus puhoatensis
